# Research on Financing Mechanism of Long-Term Care Insurance in Xiamen, China: A System Dynamics Simulation

**DOI:** 10.3389/fpubh.2021.714044

**Published:** 2021-08-16

**Authors:** Liangwen Zhang, Sijia Fu, Ya Fang

**Affiliations:** ^1^State Key Laboratory of Molecular Vaccinology and Molecular Diagnostics, School of Public Health, Xiamen University, Xiamen, China; ^2^Key Laboratory of Health Technology Assessment of Fujian Province University, School of Public Health, Xiamen University, Xiamen, China; ^3^School of Economics, Xiamen University, Xiamen, China

**Keywords:** long-term care insurance, financing mechanism, system dynamic model, sustainability, modeling and simulation

## Abstract

**Objective:** This study aimed to predict the changing trend of long-term care insurance (LTCI) funds by clarifying the linkage between revenue and expenditure and its influencing factors and to provide evidence for the establishment of a sustainable LTCI financing mechanism in China.

**Method:** We have taken Xiamen as an example, based on the data from Xiamen Special Economic Zone Yearbook and field survey. The changing trend of LTCI funds is predicted from 2020 to 2030 based on the system dynamics model (SDM) of the LTCI financing system. Also, through literature research and expert consultation, we found the intervention goals and analyzed their impact on the balance of LTCI funds.

**Results:** In the current situation, according to the forecast, the revenue and the expenditure of the LTCI funds will increase year by year from 2020 to 2030 in Xiamen, an increase of about 3.7 times and 8.8 times, respectively. After 2029, the expenditure will exceed the revenue of the LTCI funds and the balance will turn into a deficit. From the perspective of fund revenue, by adjusting the individual payment rate, government financial subsidies, and enterprise payment rate, the proportion of LTCI funds can be increased to alleviate the balance deficit under the original forecast. On the contrary, from the perspective of fund expenditure, increasing the proportion of reimbursement and the rate of severe disability will lead to an increase in fund expenditure. In this case, the balance of the funds will turn into a deficit, 7 years in advance. In addition, it was found that the severe disability rate has the greatest impact on the balance of funds.

**Discussion:** The SDM can objectively reflect the structure and the behavior of the LTCI financing system and has good applicability. By increasing the individual payment rate, government financial subsidies, and enterprise contribution rate, reasonable setting of the reimbursement ratio of nursing services, especially for the prevention of disability among the elderly, to maintain the sustainability of the funds. This study provides strong evidence for policymakers to establish a sustainable LTCI system in China.

## Introduction

China has entered a period of rapid development of population aging, facing the severe challenge of deep aging. At the end of 2019, 254 million people were aged over 60 years, accounting for 18.1% of the total population, according to the National Bureau of Statistics of China (1). It is projected that the proportion of elderly people will reach one-third by 2050, exceeding that of most EU countries (2). As the population ages, the number of disabled people also increases (3). With the increasing demand for professional nursing care for the elderly, traditional home-based care is unsustainable. The long-term care insurance (LTCI) system has emerged as an important measure to respond to the needs of social care caused by the aging of the population and to reduce the economic and care burden on the individuals and their families (4). Under the background of the change of family structure and the weakening of traditional support functions, it is critical to establish an LTCI system that suits the national conditions of China.

### LTCI in China

At present, the Chinese government is actively implementing the LTCI policy. In June 2016, the Chinese government published the “Guidance on Pilot Cities to Launch Long-Term Care Insurance.” At the end of June 2019, the LTCI scheme has been piloted in 15 cities, covering 88.54 million people, and benefiting 426,000 people (5). In September 2020, the National Medical Security Administration published the “Guidance on Expanding the Pilot Cities of the Long-Term Care Insurance” to expand the pilot program of the LTCI; 14 new LTCI pilot cities had been planned to be added to the original 15 pilot cities. The pilot period will be 2 years (6). It can be seen that the establishment and improvement of the LTCI system are the inevitable choices for China to cope with the aging population. Enrolment in LTCI is linked to the medical insurance status of individuals. China currently has two medical insurance schemes, namely, the urban employee medical insurance, covering urban residents with formal employment, and medical insurance for urban and rural residents, covering rural and urban residents without employment. The insured of LTCI must be the insured of the urban employee insurance or the medical insurance for urban and rural residents (7). In addition, according to the disability assessment criteria set by each pilot area, most of the pilot areas guarantee the long-term care (LTC) needs of the severely disabled. The main types of care are home care and institutional care, and the standard of insurance payment is about 50–70%. The operation of LTCI involves many aspects, such as financing, evaluation, service, and supervision. Among them, financing is the core of the LTCI system and is the foundation for the formation of insurance funds, which reflects the internal mechanism of financing. How to build a fair, reasonable, and sustainable financing mechanism is the primary problem when establishing an LTCI system (8).

### Previous Research

At present, the research abroad has an early start in which theoretical research and empirical studies have achieved fruitful results. Previous studies mainly focused on the financing model (9, 10), equity (11), and sustainability of financing (12), advantages and disadvantages of public and private LTCI financing (13, 14), and comparison of financing mechanisms (15–17). The quantitative studies on the financing mechanism of LTCI have a wide range of contents and mature methods. The research content includes basic nursing demand forecasting (18, 19), insurance pricing (20), policy simulation (18, 20), and cost control (21), among others, and the system dynamics model (SDM) is introduced to optimize the pension security system (22, 23). These results provide a reference for Chinese scholars.

At present, some domestic scholars focus on analyzing and summarizing the LTCI financing in OECD countries and domestic pilot cities (24, 25) and gradually expanding to financing channels (26), financing models (6), financial supply and demand forecasts (7, 27, 28), LTCI contribution rate (29, 30) and other aspects. The research methods mainly focus on the traditional logistic regression, actuarial model, International Labor Organization financing model, and so on. Few studies have analyzed the financing mechanism of LTCI from a systematic perspective. LTCI financing system involves complex social demography, health management, economics, and other multi-dimensional variables. Research showed that SDM is well-suited to address the dynamic complexity of health-related delivery systems (31). This study considered Xiamen as an example to construct an SDM of the LTCI financing system. As one of the five special economic zones in China, Xiamen is facing the risks and challenges brought by the aging population, such as the high aging rate and the average life expectancy of the population, and it is urgent to maintain the balance between the supply and demand of nursing services for the elderly (32), which is representative and typical.

Therefore, from the perspective of equity, efficiency, and sustainability, this study constructs the SDM of LTCI financing in Xiamen under the background of aging, makes a medium- and a long-term prediction of LTCI financing, which makes the prediction results more scientific and accurate, and observes the changing trend of LTCI funds in the future. Then, by adjusting the key policy indicators, such as individual payment rate, the influence of intervention goals on the financing system of LTCI was analyzed to provide evidence of the need for the establishment of a sustainable financing mechanism for multiple financial supplies.

## Materials and Methods

### Data Sources

Data for this study are taken from field research, expert consultation, the yearbook of Xiamen Special Economic Zone, and published research literature. Based on the principle of typical sampling, four representative LTCI pilot cities (Jiaxing, Shanghai, Chengdu, and Jingmen) were selected from the east, middle, and west of China to conduct the field research. The survey content mainly involves the insured population, the method and level of financing, and payment to reflect the overall construction of the LTCI system. The main data sources are listed in Table 1.

**Table 1:** Summary of main data sources.

**Parameters**	**Value**	**Unit**	**Source**
Insured population of urban employees	273.88	10,000 people	Xiamen Special Economic Zone Yearbook 2020
Per capita GDP	142,739	Yuan	Xiamen Special Economic Zone Yearbook 2020
Per capita disposable income of urban residents	55,870	Yuan	Xiamen Special Economic Zone Yearbook 2020
Insured population of urban and rural residents	148	10,000 people	Xiamen Special Economic Zone Yearbook 2020
Per capita institutional care costs^b^	30,000	Yuan	Survey data
Per capita home care costs^b^	18,000	Yuan	Survey data
Change rate of insured population of urban employees^a^	6%	Yuan	Xiamen Special Economic Zone Yearbook(2011-2020)
Change rate of insured population of urban and rural residents^a^	5.9%	Yuan	Xiamen Special Economic Zone Yearbook(2011-2020)
Change rate of per capita GDP^a^	5%	Dimensionless	Xiamen Special Economic Zone Yearbook(2000-2020)
Government per capita subsidy standard^b^	0.04%	Dimensionless	Assumed value
Change rate of per capita disposable income of urban residents^a^	8%	Dimensionless	Xiamen Special Economic Zone Yearbook(2015-2020)
Enterprise contribution rate^b^	0.04%	Dimensionless	Assumed value
Individual payment rate^b^	0.06%	Dimensionless	Assumed value
Severe disability rate^b^	0.3%	Dimensionless	Historical literature (33)/Survey data
Proportion of choosing the institutional care^b^	3%	Dimensionless	Historical literature (34)
Proportion of choosing home care^b^	97%	Dimensionless	Historical literature (34)
Reimbursement ratio^b^	70%	Dimensionless	Assumed value

a *Calculated by using data from Xiamen Special Economic Zone Yearbook*.

b *According to the actual operation and investigation of LTCI, the base of severe disability rate is the total number of insured people. The base of individual payment rate is the per capita disposable income of urban residents in 2019; and the enterprise contribution rate and government per capita financing standard base per capita GDP in 2019*.

### Key Assumptions

At present, Xiamen does not implement the LTCI system. Therefore, according to published research literature, combined with the field research, the key assumptions of this study are as follows: (a) Xiamen implemented LTCI in 2019, and the forecast time of the model is 2020-2030. (b) At present, the enrollment in LTCI is linked to the medical insurance status of individuals' in pilot cities. To ensure the fairness of the implementation of the system, it is assumed that those who participate in LTCI are insured of all medical insurance, and those who enjoy the benefits of LTCI are the severely disabled people in the insured population. (c) Funds for LTCI mainly come from premium payments, without taking into account the investment and operation income of insurance institutions. (d) Xiamen implements a compulsory social LTCI system, which raises funds through various channels such as individual, enterprise contribution and financial subsidies and establishes a diversified financing mechanism, which is not attached to the medical insurance funds. (e) The economic development is relatively stable, and the per capita GDP and wage levels maintain a certain growth rate without major fluctuations.

### Model Construction

#### System Analysis

**Figure 1:**
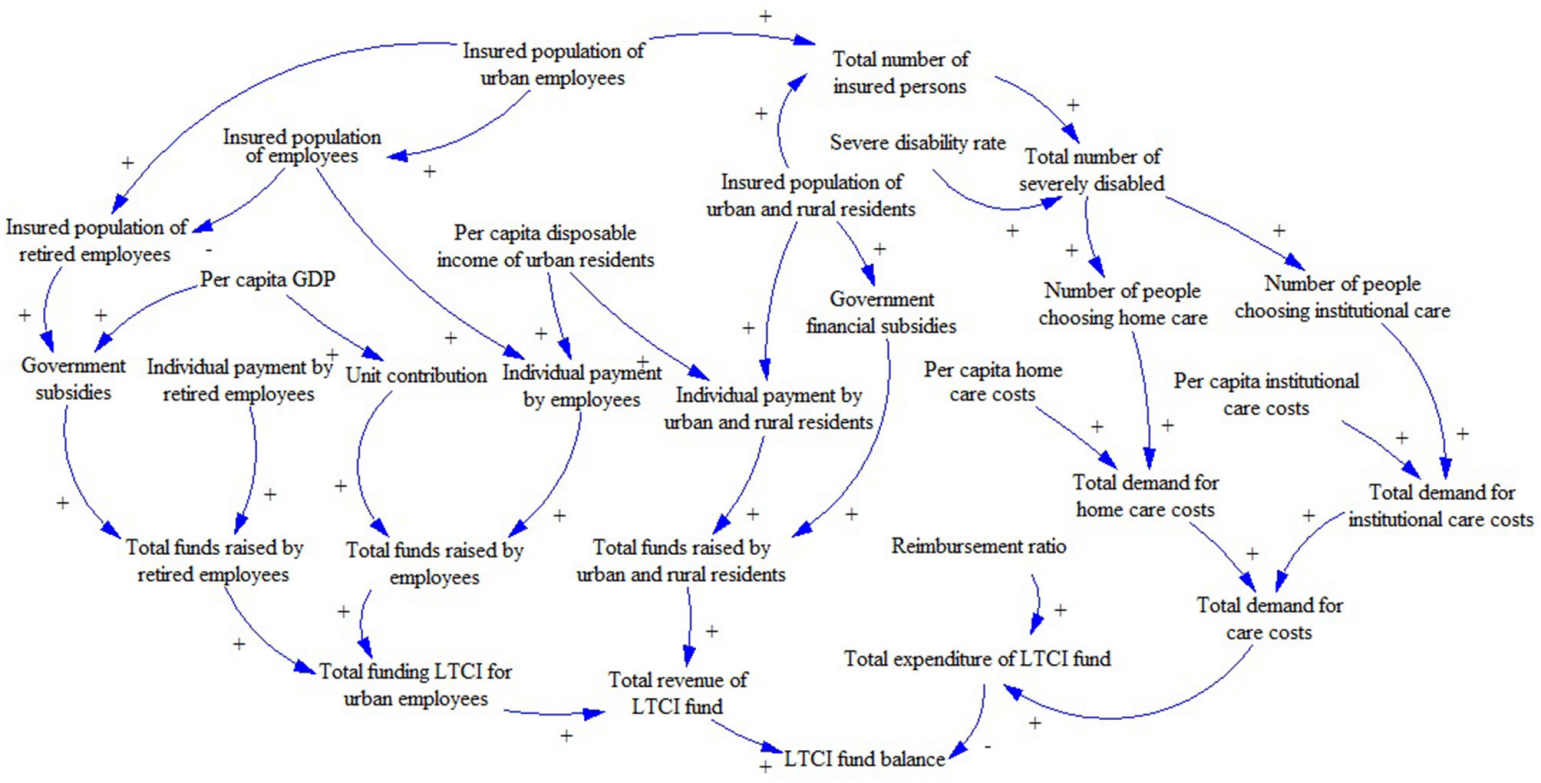
Causality diagram of LTCI financing system.

**Figure 2:**
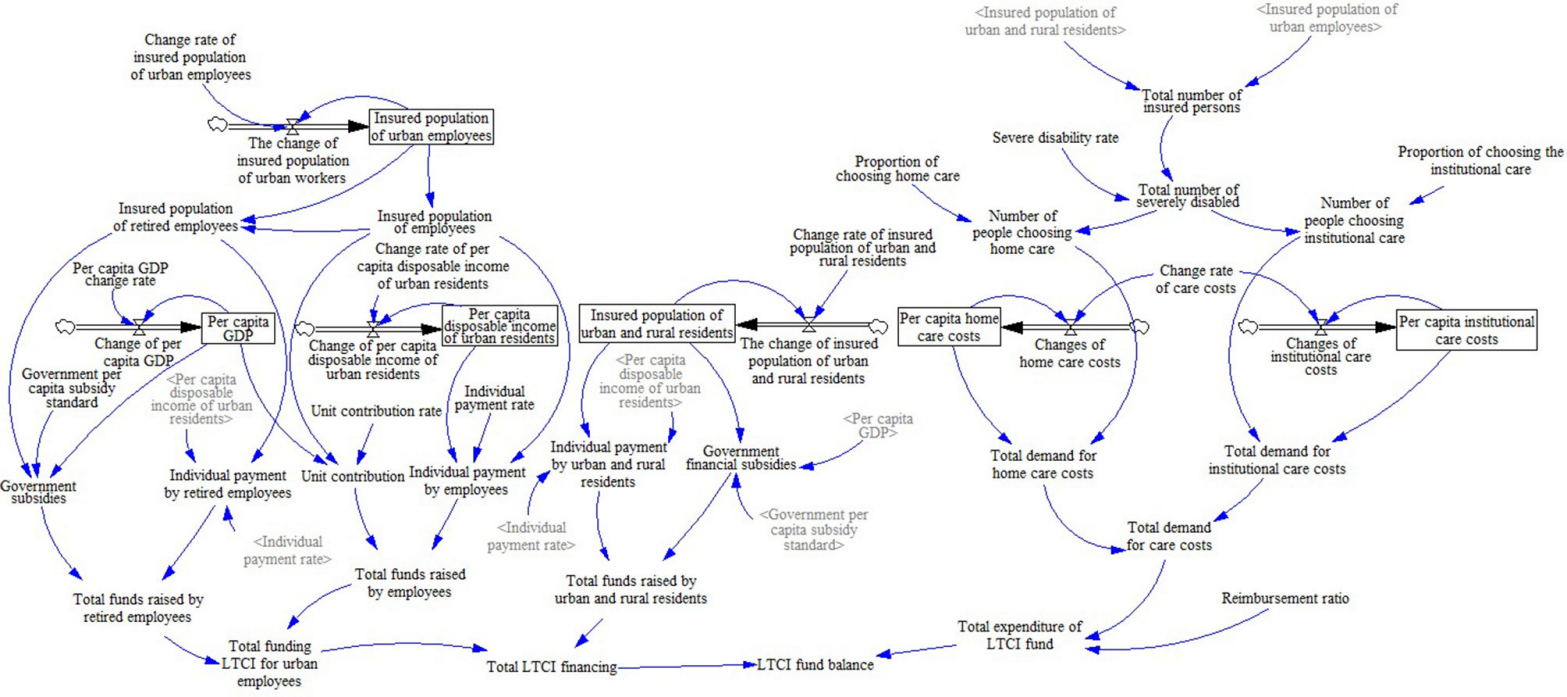
Flow diagram of LTCI financing system.

The modeling process of SDM mainly includes: (a) System subject analysis: Through collecting and analyzing the policy documents and literature related to the LTCI fund, this study identifies the stakeholders of the LTCI financing system. On this basis, the system analysis method is used to determine the main body of the system: individuals, enterprises, and the government. (b) Drawing of the causal diagram: In the SDM, the system structure is composed of the feedback loop, which shows the relationship between variables and the action path. Based on the purpose of the study and the actual operation of LTCI financing, this study makes a loop analysis on the revenue subsystem and the expenditure subsystem of the LTCI funds. The variables of the two subsystems and each subsystem are interrelated and restricted and ultimately affect the balance of the LTCI funds (Figure 1). In this figure, the arrow represents the relationship among variables, and the direction of each line shows the direction of the effect. The sign “+” dictates that the variables change in the same direction, while the sign “–” dictates that the variables change in the opposite direction. (c) Model construction: According to the causality diagram, with the help of a literature review and system analysis theory, the relationships between variables in the model are defined (Figure 2). The revenue subsystem of LTCI funds includes the revenue of urban employees, retirees, and urban and rural residents. The expenditure subsystem of LTCI funds is affected by the total demand and reimbursement ratio of LTC expenses. Through drawing the system flow diagram and establishing the structural equation, the initial value of variables refers to official statistics, and the functional relationship between the variables are determined by the social insurance actuarial method and the regression analysis method. (d) Simulation and policy optimization: Based on the operation of LTCI, combined with field survey and sensitivity analysis, this study selects five key variables that affect the revenue and the expenditure of the LTCI fund for scenario simulation.

#### Sensitivity Analysis

**Figure 3:**
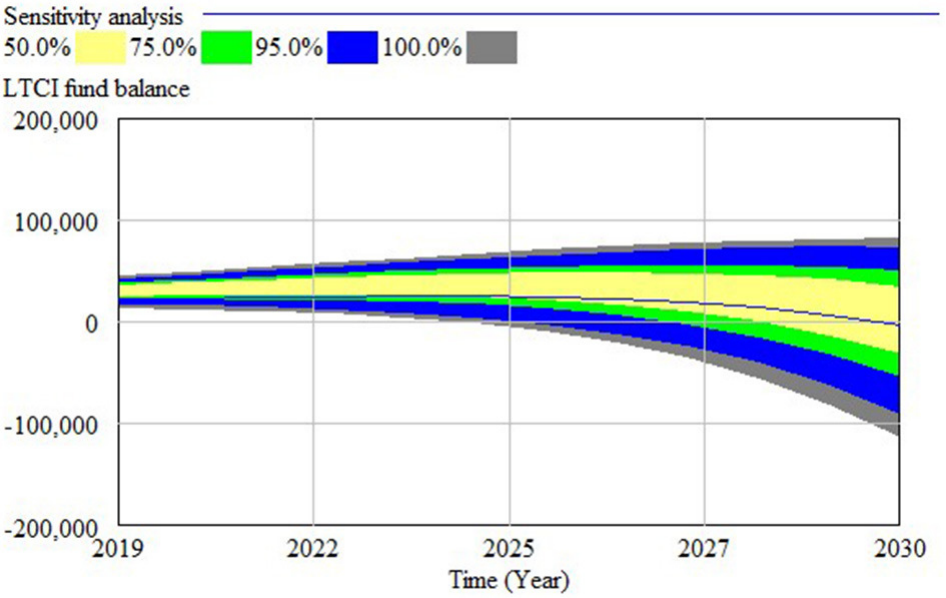
Sensitivity analysis diagram.

Sensitivity analysis determines the influence degree of parameters on the model by changing the parameters and comparing the output of the model. Based on the LTCI operation situation, combined with field investigations, we selected five key variables that affect the revenue and the expenditure of the LTCI funds for sensitivity analysis, namely, individual payment rate, government financial subsidies and enterprise contribution rates, reimbursement ratio of nursing services, and severe disability rate, to verify the influence of parameters on the balance of LTCI funds to achieve sensitivity analysis. We set the number of verifications to 200 and used random uniform distribution to verify. The results show that the LTCI fund balance has changed significantly by adjusting the parameter range (Figure 3), which provides a reference for policy intervention.

#### Historical Test

The model has passed the dimensional consistency test, the structural test, and the historical test. According to the data availability and the historical tests, the number of insured medical insurance by urban employees in population factors and the per capita disposable income of urban residents in economic factors are selected for the simulation data. By comparing the data of the medical insured population of urban employees and per capita disposable income of urban and rural residents in the yearbook of Xiamen Special Economic Zone (2012-2019), the historical test of the model was carried out. The average error between the actual data and the simulation data is 0.67% (Table 2). Within a reasonable range, the model is highly fitting, effective, and reasonable.

**Table 2:** Historical test.

**Year**	**Medical insured population of urban employees**			**Per capita disposable income of urban residents**		
	**Actual data/10,000 people**	**Simulation data/10,000 people**	**Error rate/%**	**Actual data/10,000 people**	**Simulation data/10,000 people**	**Error rate/%**
2012	182.15	180.7244	−0.78265	37,576	38382.08	−2.1452
2013	192.26	191.4216	−0.43608	41,360	36,772	11.09284
2014	203.64	199.4962	−2.03487	39,625	39539.296	0.216288
2015	212.23	209.7704	−1.15893	42,607	42923.712	−0.74333
2016	223.16	228.185	2.251748	46,254	46417.632	−0.35377
2017	242.75	241.956	−0.32709	50,019	50484.128	−0.9299
2018	257.4	257.4472	0.018337	54,401	51847.36	4.694105

## Results

The SDM can objectively reflect the structure and the behavior of the LTCI financing system and has good applicability. In this section, first, we analyze the changes in the revenue, expenditure, and balance of the LTCI fund in the current situation and, second, we analyze the future trend of the fund by changing the five key variables that affect the balance of the LTCI fund. Finally, by comparing the impact of these variables on the fund balance, the corresponding conclusions are drawn.

### Analysis of the Current Situation

**Figure 4:**
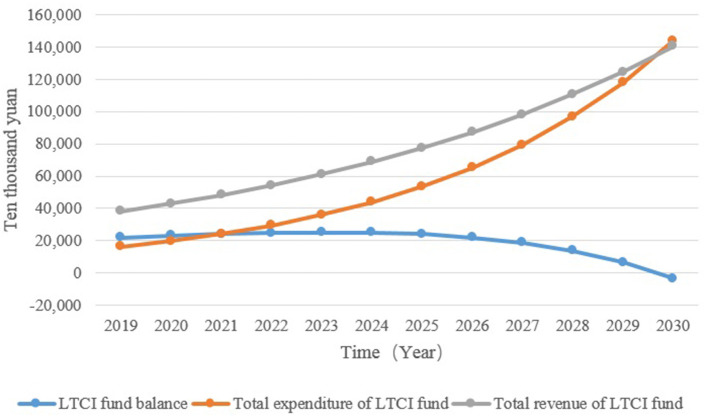
The revenue, expenditure, and balance of LTCI funds.

Figure 4 and Table 1 show the revenue, expenditure, and balance of the LTCI funds from 2020 to 2030. The revenue and the expenditure of the LTCI funds show an increasing trend year by year, an increase of about 3.7 times and 8.8 times, respectively. After 2029, the expenditure of the LTCI funds will exceed the revenue of funds, and the balance of funds will increase first and then decrease; it will reach –¥34.05 million in 2030.

### Policy Intervention

Based on the operation of LTCI, combined with field investigation, this study selected five key variables affecting the revenue and the expenditure of LTCI funds, including individual payment rate, reimbursement ratio of nursing services, the rate of severe disability, government financial subsidies, and enterprise contribution rates. Key variables were adjusted to conduct scenario simulations, and the changing trends of LTCI funds revenues, expenditures, and balances were compared under different intervention programs.

#### Adjust Individual Payment Rate

To ensure the fairness of the implementation of the system, the base of the individual payment rates of urban employees and urban and rural residents is the per capita disposable income of urban residents. Also, to decrease the burden of the individual payment, according to the practice of the pilot area, the proportion of simulated set value was set between 0.06 and 0.1%, which is about ¥30–55 (based on the per capita disposable income of urban residents in 2019).

Test1-1: individual payment rate = 0.06%
Test1-2: individual payment rate = 0.08%
Test1-3: individual payment rate = 0.1%

**Figure 5:**
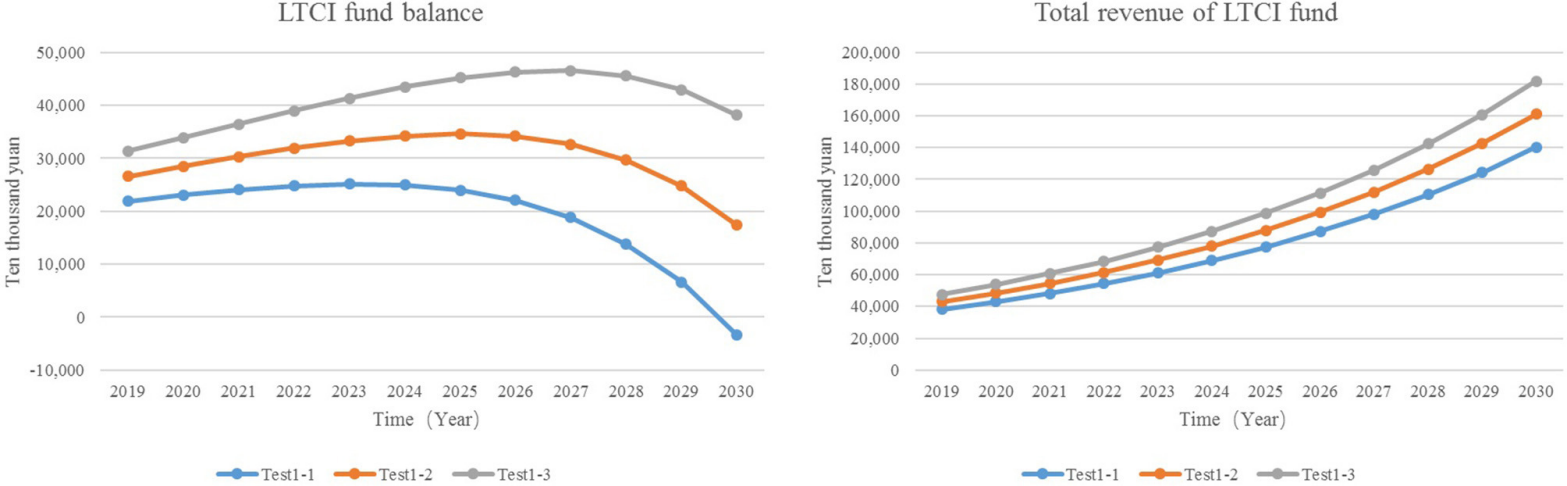
The revenue and balance of LTCI funds.

The results show that in comparison with Test1-1, the revenue of the LTCI funds of Test1-3 increased by ¥416 million to ¥1.819 billion, and the balance increased by 415.75 million to ¥381.7 million. Test1-1 will turn into a deficit in the balance of the LTCI funds in 2030, and after raising the individual payment rate (Test1-2 and Test1-3), the balance of funds did not turn into a deficit in the simulated years (Figure 5). Therefore, the increase in individual payment rates can increase the revenue of the LTCI funds and delay the year when the balance of funds turns into a deficit.

#### Adjust the Reimbursement Ratio of Nursing Services

All of the policy documents pointed out “differentiated treatment guarantee policies are formulated according to the level of care and service delivery methods, and the payment level of funds is generally controlled at about 70% for LTCI expenditure that meets the requirements.” According to the practice in the pilot area, the reimbursement ratio of nursing services is about 50–90%. Therefore, this study sets the simulation value of the reimbursement ratio as 50, 60, 70, 80, and 90%.

Test2-1: Reimbursement ratio = 50%
Test2-2: Reimbursement ratio = 60%
Test2-3: Reimbursement ratio = 70%
Test2-4: Reimbursement ratio = 80%
Test2-5: Reimbursement ratio = 90%

**Figure 6:**
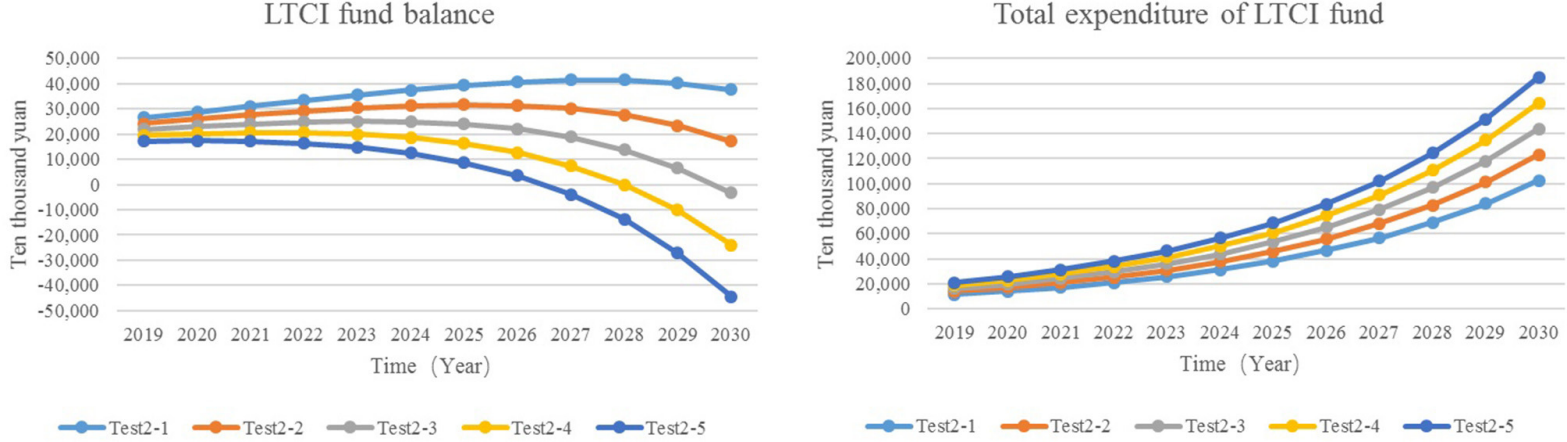
The expenditure and balance of LTCI funds.

The results showed that, with the increase in the reimbursement ratio of nursing services, the expenditure of LTCI funds also increased. In comparison with Test2-1, the expenditure of the LTCI funds in Test2-5 increased by ¥822 million to ¥1.848 billion in 2030, and the balance of funds turns into a deficit earlier than in other situations (Figure 6). Therefore, the increase of the reimbursement ratio will increase the expenditure of the LTCI funds and accelerate the deficit of the balance.

#### Adjust Government Financial Subsidies and Enterprise Contribution Rates

To ensure the fairness of the implementation of the system, the base of government financial subsidies and enterprise contribution rates are both per capita GDP, and the two values are equal. The proportion of the simulation set value is between 0.04 and 0.07%, that is, about ¥55–100 (based on the per capita GDP in 2019).

Test3-1: government financial subsidies and enterprise contribution rates = 0.04%.
Test3-2: government financial subsidies and enterprise contribution rates = 0.05%.
Test3-3: government financial subsidies and enterprise contribution rates = 0.06%.
Test3-4: government financial subsidies and enterprise contribution rates = 0.07%.

**Figure 7:**
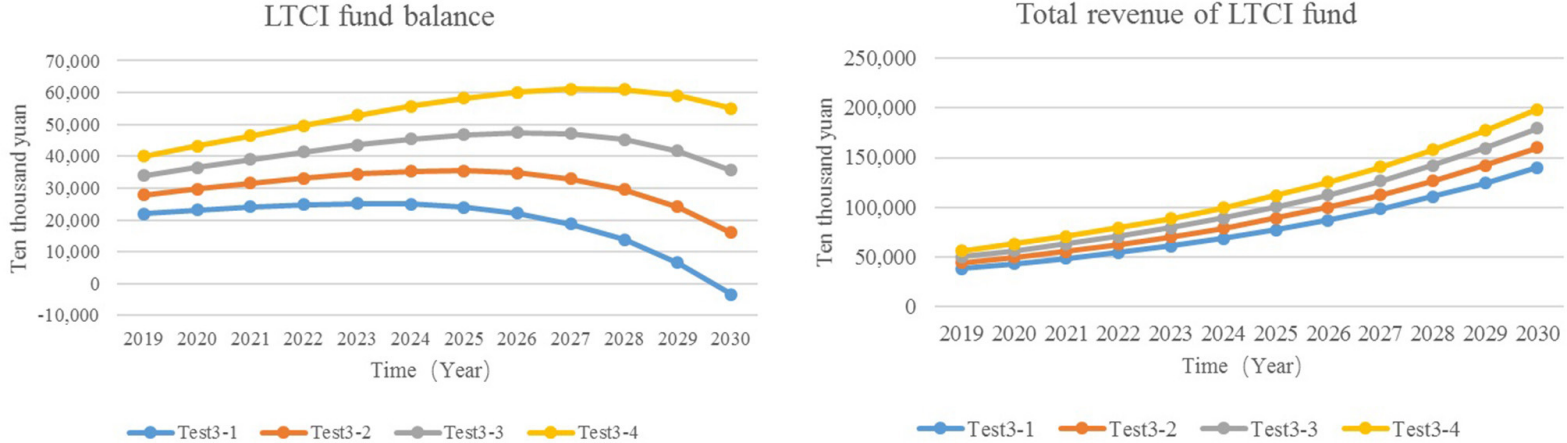
The revenue and balance of LTCI funds.

The results showed that in comparison with Test3-1, the revenue of the LTCI funds of Test3-4 increased by ¥584 million to ¥1.87 million, and the balance increased by ¥584.45 million to ¥550.4 million. Test3-1 showed a deficit in the balance of the LTCI funds in 2030. After raising the enterprise contribution rate and the per capita financing standard of the government (Test3-2, Test3-3, and Test3-4), the balance of funds did not turn into a deficit in the simulated years (Figure 7). It can be seen that raising the financing standard of enterprises and government subsidies can increase the revenue of the LTCI funds and postpone the year when the balance begins to decline.

#### Adjust the Rate of Severe Disability

All of the policy documents pointed out that the LTCI system takes the insured who has been in a state of disability for a long time as the protection object, focusing on the basic life care of severely disabled people and the medical care closely related to basic life and other expenses. At the beginning of the system, limited resources should be used on the people who are most in need of protection, which not only does not cause a waste of resources but also does not cause excessive pressure on enterprises and individuals to pay fees (35). At present, the beneficiaries of the LTCI in pilot cities of China are mainly severely disabled elderly, and the assessment criteria for disability are mainly based on the Barthel index. According to the practice in the pilot area, due to the slightly different selection of disability assessment criteria, the measured value and real value of the severe disability rate are about 0.3 and 0.5%. Therefore, in this study, the simulation value of the severe disability rate is set to 0.3, 0.4, and 0.5%.

Test4-1: The rate of severe disability = 0.3%.
Test4-2: The rate of severe disability = 0.4%.
Test4-3: The rate of severe disability = 0.5%.

**Figure 8:**
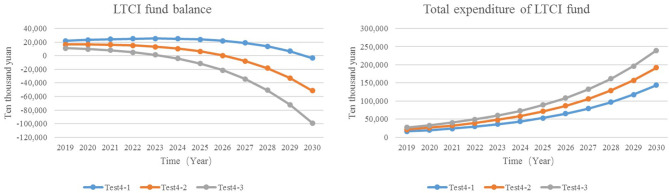
The expenditure and balance of LTCI funds.

The results showed that the total expenditure of Test4-3 is ¥958 million higher than that of Test4-1 to 2030, reaching ¥2.395 billion (Figure 8). Increasing the rate of severe disability, the balance of the LTCI funds turns into a deficit in advance. It can be seen that the lower the severe disability rate is, the lower the expenditure of LTCI funds can be reduced, and the year when the balance presents deficit can be delayed.

#### Comparison of Intervention Effect

Further analyze the differences in the impact of adjusting individual payment rates, reimbursement ratio of nursing services, severe disability rates, enterprise contribution rates, and government per capita financial subsidies on fund balances. Taking the balance of the LTCI funds as the observation index, the individual payment rate, reimbursement ratio, enterprise contribution rate, and government per capita financial subsidies increase by 10% under the condition of other conditions unchanged.

It can be seen from Table 3 that when the individual payment rate increases by 10%, the change range of the balance of the LTCI funds from 2020 to 2030 is ~8.91–54.37%; when the proportion of reimbursement increases by 10%, the change range of fund balance from 2020 to 2030 is ~-311.42 to −10.16%; when the enterprise contribution rate and the government per capita financial subsidies increase by 10%, the change range of the fund balance from 2020 to 2030 is ~-144.37 to 1.13%; finally, when the increase rate of severe disability is 10%, the change range of fund balance from 2020 to 2030 is ~-653.94 to −21.39%. It can be seen that the effects of the four intervention goals on the balance of LTCI funds are different. The effects on the balance of the LTCI funds from high to low are the rate of severe disability, the reimbursement ratio, the rate of enterprise contribution, the government per capita financial subsidies, and the rate of individual payment rates.

**Table 3:** Percentage change of the long-term care insurance funds balance under different intervention schemes (%).

**Year**	**Based on the original research hypothesis, increase 10%, respectively**			
	**Individual payment rates**	**Government financial subsidies, and enterprise contribution rate**	**Reimbursement ratio**	**Severe disability rate**
2019	8.91	1.13	−10.16	−21.39
2020	9.85	0.18	−12.12	−25.46
2021	11.06	−1.09	−14.7	−30.8
2022	12.79	−2.84	−18.23	−38.21
2023	15.36	−5.27	−23.31	−49.01
2024	19.36	−9.32	−31.56	−66.3
2025	26.69	−16.64	−46.73	−98.17
2026	44.55	−34.58	−83.84	−176.08
2027	154.37	−144.37	−311.42	−653.94
2028	95.96	−105.97	−207.69	−436.09
2029	35.29	−45.26	−81.94	−172.04
2030	21.12	−31.12	−52.61	−110.43

## Discussion

The financing system of LTCI involves complex social demography, health management, economics, and other multi-dimensional variables. Regarding it as a large system, its main body includes the government, enterprises, insured persons, and nursing institutions, among others, which also contains multiple subsystems and their influencing factors that affect and restrict each other. This study is based on the SDM of Xiamen LTCI financing to simulate policy intervention strategies. Without any intervention, the revenue and the expenditure of the LTCI funds show an increasing trend year by year, an increase of about 3.7 times and 8.8 times, respectively. After 2029, the expenditure of the LTCI funds exceeds the revenue, and the accumulated balance will turn into a deficit, reaching –¥34.05 million in 2030, at which time the LTCI funds will be insolvent. The results show that, if no intervention measures are taken, the revenue and the expenditure of the LTCI funds will face the risk of imbalance. By predicting the future development trend of LTCI funds, it reflects the necessity and urgency of improving the LTCI financing system and establishing a unified LTCI financing mechanism.

This study introduced the established LTCI financing SDM as an experimental platform to simulate policy interventions and judged the effects and impact of various strategies by observing changes in system behavior. Aiming at the key variable of LTCI funds, first, by setting the individual payment rate of LTCI participants at 0.08–0.1%, it was found that increasing the individual payment rate can delay the balance of funds turn into a deficit; second, the enterprise contribution rate and government per capita financing standard are adjusted to 0.04–0.07%. The results showed that the balance of funds does not turn into a deficit in the simulation period when the government financial subsidies and enterprise contribution rate are increased. By adjusting the pooling funds and personal accounts of basic medical insurance, the LTCI financing mechanism undertaken by individuals, enterprises, and governments can be constructed, without increasing the social security payment burden of the enterprises and individuals (36). With the exploration and improvement of the system, it was recommended to increase the payment capacity of the LTCI funds promptly and further increase the government financial subsidies for urban and rural residents in LTCI. At this stage, the people who enjoy the LTCI are the elderly group. To ensure the rationality and fairness of the system, it is recommended to implement a paying policy for urban retired employees. Without causing pressure on the payment of retirees and the government, the payment of premiums by retired employees can increase the revenue of the LTCI funds and maintain its stability and sustainability (37).

In addition, the policy documents all pointed out “differentiated treatment guarantee policies are formulated according to the level of care and service delivery methods, and the funds' payment level is generally controlled at about 70% for LTCI expenditure that meets the requirements (5, 6)”. According to the practice of pilot areas, the reimbursement ratio of nursing services is adjusted to be about 50–90%. The results showed that, as the reimbursement ratio increased, the expenditure of the LTCI funds also increased, accelerating the deficit in the balance of funds. Therefore, to maintain the sustainability of the funds, the initial payment standard of nursing service should not be too high and should be reasonably determined according to the principle of “determining expenditure by revenue.” In the initial stage of the implementation of the system, through timely analysis of the service content and payment standards formulated in the previous period, the actual application of the service content and payment standards are continuously adjusted according to the actual situation. In the later stage of the implementation of the system, passive protection after the event can be transformed into an active comprehensive protection of “prevention + compensation,” which not only guarantees the sustainability of system operation but also helps to build a healthy aging society (38, 39).

Finally, according to the practice of pilot areas, due to the slightly different selection of disability assessment criteria, the adjusted severe disability rate is about 0.3–0.5%. The results show that increasing the severe disability rate will lead to the deficit of the LTCI balance of funds ahead of time. In comparing the effect of four intervention targets on the balance of the fund, it was found that the severe disability rate has the greatest impact on the fund balance. At present, the domestic assessment tools for disability levels are mainly based on the Barthel index, which measures basic daily living ability. The content of the disability assessment is relatively single, only measuring self-care ability, and the disability assessment is not comprehensive enough. If only a single Barthel index scale is used for disability assessment, it may lead to a high disability rate, which is not conducive to maintaining the stability and sustainability of the LTCI funds. Therefore, combined with the current practice in pilot areas, it is suggested that the following points should be considered in the construction of disability assessment tool: (a) at present, China has not yet formed a unified national LTCI system, and the selection of disability assessment content should match with the local LTCI service delivery capacity and scientifically judge the degree of disability; (b) developing the corresponding disability assessment information management system and using the information system to complete the assessment work, which can avoid subjective and human factors in the assessment; and (c) relying on the local medical institutions at all levels and establishing a database of evaluation experts to ensure fairness and justice in the evaluation (40).

Under the current social background, foreign experience shows that it is urgent to build an LTCI system with social insurance as the main body, financial subsidies as the support, and commercial insurance as the supplement. At the same time, policymakers should establish a multi-dimensional and dynamic financing mechanism shared by individuals, enterprises, and government (41). As far as China is concerned, it has become a trend to design LTCI as an independent financing insurance, which is parallel to other social insurance. However, few studies have analyzed the financing mechanism of LTCI from a systematic perspective. Therefore, based on the field research, this study constructs the financing mechanism of the individuals, enterprises, and the government, which can provide the basis for the further implementation and sustainable development of the system and also the reference for other developing countries to establish a sustainable LTCI system (42). Part of the data is based on field investigation and expert consultation conducted in four representative LTCI pilot cities, Jiaxing, Shanghai, Chengdu, and Jingmen, which increased the reliability of the model and enriched the quantitative research on the LTCI system. It provides theoretical support for the construction and optimization of the Xiamen LTCI financing mechanism. However, there are still some limitations to our study. First, due to the lack of relevant data, the parameters are set using the estimation method and lack of dynamic, such as severe disability rate, which may affect the accuracy of the model prediction (43). Second, this study mainly takes Xiamen as an example, lacking a comprehensive study on the national LTCI system, but it provides ideas for the study of the national LTCI financing mechanism. Therefore, in the future, we will carry out research nationwide and set some parameters accurately to provide a more valuable reference for the development and improvement of the LTCI system in China.

## Conclusions

In summary, the SDM can objectively reflect the structure and the behavior of the LTCI financing system and has good applicability. The results show that the revenue and the expenditure of the LTCI funds display an increasing trend year by year. By increasing the individual payment rate, government financial subsidies, and enterprise contribution rate, reasonable setting of the reimbursement ratio of nursing services, especially the prevention of the elderly disability, to maintain the sustainability of the funds. Therefore, first, it is possible to build an LTCI financing mechanism borne by individuals, enterprises, and government, to further increase the government financial subsidies to urban and rural residents, to implement the payment system for retired employees, to increase the revenue of LTCI funds, and to improve the payment ability of the funds in the future. Second, policymakers should build a fair and effective disability assessment system according to the actual situation of the pilot areas to scientifically judge the degree of disability. In addition, a reasonable standard system of treatment protection should be constructed in accordance with the principle “expenditure is determined by revenue, the balance between revenue and expenditure” to maintain the stability and sustainability of the LTCI funds.

## Data Availability Statement

The original contributions presented in the study are included in the article/supplementary material, further inquiries can be directed to the corresponding author/s.

## Author Contributions

LZ contributed to the study conception, design, and drafted the manuscript. SF participated in the statistical analysis and drafted the manuscript. YF supervised and revised the manuscript. All authors read and approved the final manuscript.

## Conflict of Interest

The authors declare that the research was conducted in the absence of any commercial or financial relationships that could be construed as a potential conflict of interest.

## Publisher's Note

All claims expressed in this article are solely those of the authors and do not necessarily represent those of their affiliated organizations, or those of the publisher, the editors and the reviewers. Any product that may be evaluated in this article, or claim that may be made by its manufacturer, is not guaranteed or endorsed by the publisher.
